# Identification of two strains of *Paenibacillus* sp. as indole 3 acetic acid-producing rhizome-associated endophytic bacteria from *Curcuma longa*

**DOI:** 10.1007/s13205-012-0086-0

**Published:** 2012-09-11

**Authors:** Agnes Joseph Aswathy, B. Jasim, Mathew Jyothis, E. K. Radhakrishnan

**Affiliations:** School of Biosciences, Mahatma Gandhi University, Priyadarshini Hills, Kottayam, 686 560 Kerala India

**Keywords:** Endophytic bacteria, Indole 3 acetic acid, HPLC, *Paenibacillus* sp., 16S rDNA sequencing

## Abstract

*Curcuma longa* is well known for its use as spice and medicine. The remarkable feature of the plant is the presence of rhizome, which provides an interesting habitat for association by various groups of bacteria. Some of these associated endophytic bacteria can have growth-promoting effects. In the current study, two species of endophytic *Paenibacillus* has been identified from the rhizome as indole 3 acetic acid producers. These isolates can thus have potential growth-regulating effect in rhizomes.

## Introduction

Endophytes are microorganisms that live in the internal tissues of plants without altering the normal functioning of the tissues (Stone et al. 2000). These microorganisms are unique in their adaptations to specific chemical environment of the host plant. Some of these microorganisms have the molecular machinery for the synthesis of host plant-specific compounds and are thus considered as an unexplored source of natural products (Strobel 2003). The host plants are also benefited by the endophyte-mediated production of plant growth regulators, resistance to diseases and also endophyte-assisted phytoremediation.

*Curcuma longa* is a rhizomatous plant of the family *Zingiberaceae*. Its rhizome is well known for its medicinal properties: anti-inflammatory, antiulcerogenic and antitumor activities (Miquel et al. 2002). The active constituents of turmeric rhizome include curcumin and various volatile oils, including tumerone, atlantone and zingiberone. In addition to its chemical complexity, rhizome is biologically interesting because of its ability to stay viable for a long time under adverse conditions (Ramirez-Ahumada Mdel et al. 2006). At the same time, the rhizome is least explored in terms of its biochemical processes at various stages of its development. The close contact of the rhizome with the soil largely favors the association by various communities of microorganisms as endophytes. These associated microbes can have a remarkable role in rhizome physiology and can have varying roles in regulating plant growth.

Microbial association as endophytes has been reported from a variety of plants. Variations in the endophytic community of plants are also known with regard to the plant source, age, type of tissue, season of sampling and environment. Usually, the concentration of the endophytic bacteria is higher at the root than at shoot tissue. A large variety of plant species are shown to be endophytically associated with bacteria such as *Pseudomonas*, *Bacillus*, *Azospirillum*, etc. (Chanway 1996). Several genera of endophytic bacteria have been isolated from different plants, including *Aerobacter*, *Aeromonas*, *Agrobacterium*, *Chryseomonas*, *Curtobacterium*, *Enterobacter*, *Erwinia*, *Flavimonas* and *Sphingomonas* (Sturz et al. 1997). Studies also showed the presence of endophytic diazotrophs, *Acetobacter diazotrophicus*, *Herbaspirillum* sp., *Burkholderia* sp., *Enterobacter* sp. and *Klebsiella* sp., associated with sugarcane plants (Boddey et al. 2003). So, by considering the remarkable features of turmeric rhizome, the presence of a diverse and even specific bacterial association can well be expected.

The functional roles of endophytes are studied only from limited cases, though their presence has been reported from a wide variety of plants. The production of plant growth-promoting molecules like indole 3 acetic acid (IAA) is an important contribution of endophytic microorganisms (Spaepen et al. 2007). IAA is synthesized mainly through a coupled transamination and decarboxylation of l-tryptophan (l-Trp) by microorganisms and plants (Bhavdish et al. 2003). IAA can stimulate both rapid responses such as cell elongation and long-term responses such as cell division and differentiation of plants tissues (Taghavi et al. 2009). Previous studies have shown that IAA plays a major role in controlling various physiological processes in plants including apical dominance, tropism, shoot elongation and root initiation. Due to its importance in plants, endophyte-mediated production of IAA has gained a great deal of attention.

In the case of *Curcuma longa*, the presence of rhizome is expected to provide a specialized habitat for the association of a diverse group of bacteria with potential impact on plant growth. This makes studies on isolation and characterization of endophytic bacteria from turmeric much more interesting and informative. In the current study, two endophytic *Paenibacillus* sp. were isolated from turmeric rhizome and both of them were found to have the ability to produce IAA as confirmed by HPLC analysis.

Rhizomes of turmeric (*Curcuma longa*) collected from Navajyothisree Karunakara Guru Research Centre for Ayurveda and Siddha, Uzhavoor, Kottayam were used as the source for isolating endophytic bacteria. The rhizomes were cleaned with tap water to remove soil and were made into 1–2 cm long pieces. These were further treated with Tween 80 for 10 min with vigorous shaking and followed by washing with distilled water several times to remove Tween 80. The samples were then dipped in 70 % ethanol for 1 min and further treated with 1 % sodium hypochlorite for 10 min. The samples were then washed several times with sterilized distilled water and the final wash was spread onto a nutrient agar plate (g/L; peptone 5, beef extract 2, yeast extract 3, sodium chloride 5 and agar 18, pH 7.0) as control. For the isolation of endophytic bacteria, the outer surface of the sterilized plant material was trimmed and placed onto nutrient agar plates. Nutrient agar is one of the commonly used media for the isolation of endophytic bacteria (Aravind et al. 2009). All plates including the control were incubated at room temperature for 5 days and observed periodically for bacterial growth. Those experiments in which bacterial growth was observed in the control plate were completely discarded. Morphologically distinct colonies as identified by colony characters were selected, purified and used for further studies.

For the molecular identification, genomic DNA was isolated from the bacterial isolates and used as template for PCR. Genomic DNA isolation of the obtained bacterial strains was conducted as per the method described by Ausubel et al. (1995). The isolates were first cultured overnight in Luria–Bertani broth and the cells were harvested from 1.5 mL culture by centrifugation. The harvested cells were resuspended in 567 μL of TE buffer and lysed using 30 μL of 10 % SDS and 3 μL of 20 mg/mL proteinase K. The mixture was then incubated for 1 h at 37 °C. The lysate was mixed thoroughly with 100 μL of 5 M sodium chloride and 700 μL chloroform:isoamyl alcohol (24:1) and centrifuged at 8,000 rpm for 10 min. After centrifugation, the aqueous layer was transferred without disturbing the interface to a fresh tube and equal volume of isopropanol was added. This was then inverted several times and centrifuged at 8,000 rpm for 10 min. The pellet was washed in 70 % ethanol (v/v) and air dried at room temperature. The dried DNA pellet was resuspended in 100 μL TE buffer and visualized in 0.8 % agarose gel (w/v). Primers used for the amplification of part of 16S rDNA was 16SF (5′-AgA gTT TgA TCM Tgg CTC-3′) and 16SR (5′-AAg gAg gTg WTC CAR CC-3′) and was selected based on a previous report of Chun and Goodfellow (1995). PCR was carried out in a 50 μL reaction volume containing 50 ng of genomic DNA, 20 pM of each primer, 1.25 units of Taq DNA polymerase (Bangalore Genei), 200 μM of each dNTPs and 1× PCR buffer. PCR was carried out for 35 cycles in a Mycycler™ (Bio-Rad, USA) with an initial denaturation at 94 °C for 3 min and cyclic denaturation at 94 °C for 30 s, annealing at 58 °C for 30 s and extension at 72 °C for 2 min with a final extension of 7 min at 72 °C. The PCR product was checked by agarose gel electrophoresis, purified and further subjected to sequencing. The sequence data was checked for similarity analysis with BLAST program (Zhang et al. 2000). The phylogenetic analysis of the 16S rDNA sequence of the isolates obtained was carried out with MEGA 5 using neighbor-joining method with 1,000 bootstrap replicates (Tamura et al. 2011).

For screening IAA production, the bacterial isolates were inoculated into 20 mL of nutrient broth supplemented with 0.2 % (v/v) of l-tryptophan and incubated for 10 days at 28 °C. After incubation, the culture was centrifuged at 3,000 rpm for 20 min to collect the supernatant. Initially, 1 mL of supernatant was mixed with 2 mL of Salkowski reagent and the tubes were incubated in dark as described earlier (Rahman et al. 2010). The development of red color was observed after 1 h of incubation. Uninoculated growth medium was used as negative control. The isolates positive in the color reaction were further inoculated into 200 mL of nutrient broth supplemented with 0.2 % (v/v) of l-tryptophan and incubated for 10 days at 28 °C. After incubation, the supernatant was collected by centrifugation at 3,000 rpm for 20 min. The supernatant was then acidified to pH 2.5–3.0 with 1 N HCl and extracted twice with equal volume of ethyl acetate. The extracted ethyl acetate fraction was vacuum dried in a rotary evaporator at 40 °C. The dried powder was dissolved in 1 mL of methanol (MeOH) and stored at −20 °C. Crude extracts made from the isolates were spotted on a thin layer chromatographic (TLC) plate (Silica gel Gf 254, thickness 0.25 mm) along with standard IAA as control. The solvent system *n*-butanol:ethyl acetate:ethanol:water (3:5:1:1) was used as the mobile phase. The plates were developed by spraying paradimethyl aminobenzaldehyde reagent and incubated at 50 °C for 1 h. The *R*_f_ values of the extracts were compared with those of the standard for identification as the methods explained by Chaiharn and Lumyong (2011). Further confirmation of the presence of IAA in the methanolic extract of culture supernatant was conducted by reverse-phase high pressure liquid chromatography (HPLC) analysis on a Supelcosil LC-18 column with a flow rate of 1 mL min^−1^ as described by Jensen et al. (1995). Elution was performed with a mixture of H_2_O and MeOH (60:40), both containing 0.5 % acetic acid. Elution was monitored at 280 nm by a Shimadzu UV–Vis detector model SPD 10A.

The surface sterilization procedure was quite satisfactory for isolation of endophytic bacteria as no growth appeared on the control plate. Also, bacterial growth was obtained in the nutrient agar plates which were inoculated with the rhizome piece. Based on the distinct colony characteristics, two isolates were obtained from the rhizome of turmeric and named as ClB1 and ClB2. The colony of ClB1 was small, pinpoint and yellow pigmented, and that of ClB2 was small, lobate margined and cream colored.

**Fig. 1:**
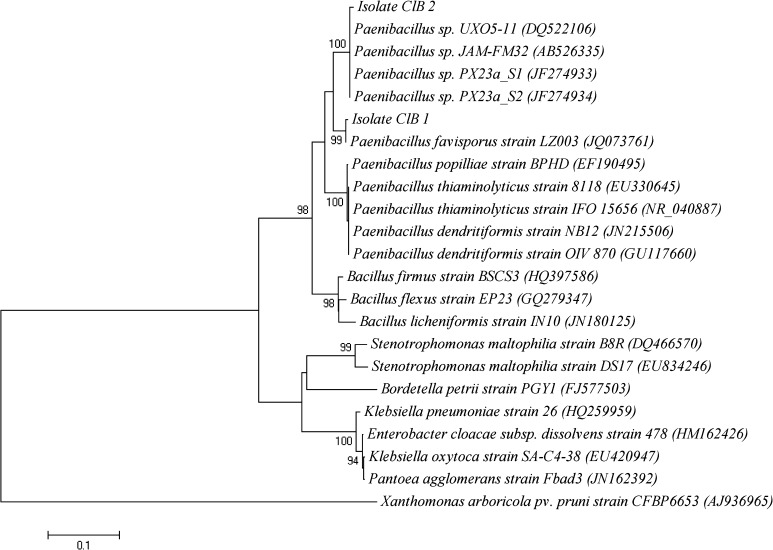
Phylogenetic analysis of 16S rDNA sequences of the bacterial isolates (ClB1 and ClB2) from *Curcuma longa* along with the sequences from NCBI. The analysis was conducted with MEGA5 using neighbor-joining method

Molecular identification of the isolates was done by the sequencing of 16S rDNA gene. The presence of 1,500 bp product as observed by agarose gel electrophoresis confirmed the amplification of 16S rDNA gene. The PCR product was gel eluted, sequenced and the sequence data were submitted to NCBI under the accession numbers (JN835217 and JN835218). As the 16S rDNA gene sequence provides accurate grouping of organism even at subspecies level, it is considered as a powerful tool for the rapid identification of bacterial species (Clarridge 2004). The sequence data thus obtained was subjected to BLAST analysis. The sequence similarity analysis of 16S rDNA sequence of ClB1 and ClB2 showed its maximum identity of 99 % to *Paenibacillus favisporus* and *Paenibacillus* sp., respectively. So the isolates ClB1 and ClB2 can be considered as strains coming under the *Paenibacillus* genus. The phylogenetic analysis of 16S rDNA sequence of the isolates along with the sequences retrieved from the NCBI was carried out with MEGA 5 using the neighbor-joining method with 1,000 bootstrap replicates. The result of phylogenetic analysis showed distinct clustering of the isolates and confirms the results of the sequence similarity analysis (Fig. 1).

Endophytic *Paenibacillus* sp. have been found associated with various plants like *Pinus* sp. and *Coffea arabica* (Bent and Chanway 2002; Vega et al. 2005). The observations of Scherling et al. (2009) confirmed experimentally the association of *Paenibacillus* sp. with the root of in vitro grown poplar plants. However, the information on the presence of two species of *Paenibacillus* associated with *Curcuma longa* as endophyte is novel. This highlights the significance of the isolates obtained in the study.

**Fig. 2:**
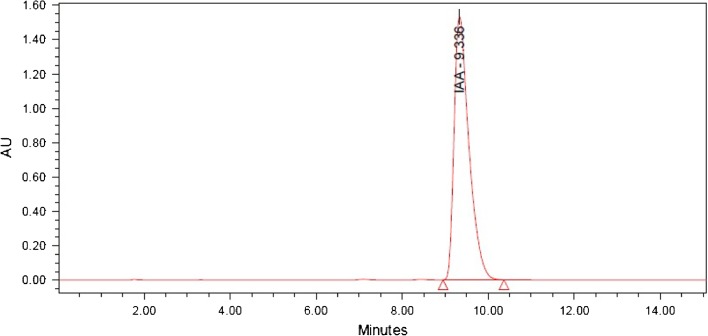
HPLC chromatogram for IAA standard carried out using water and methanol, both containing 0.5 % acetic acid in the ratio 60:40 on reversed phase C18 column with a flow rate 1 mL min^−1^ and detected under UV at 280 nm

**Fig. 3:**
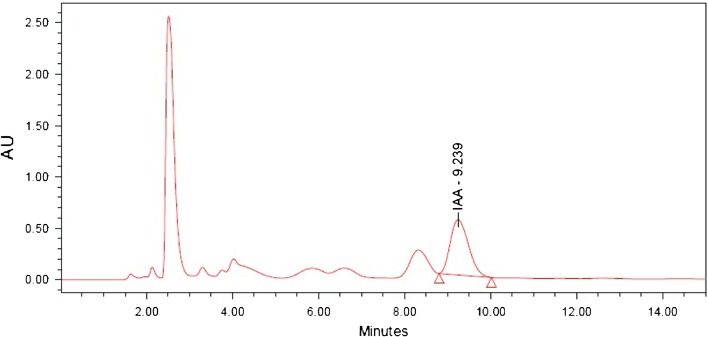
HPLC chromatogram for the extract from isolate ClB1 carried out using water and methanol, both containing 0.5 % acetic acid in the ratio 60:40 on reversed phase C18 column with a flow rate 1 mL min^−1^ and detected under UV at 280 nm

**Fig. 4:**
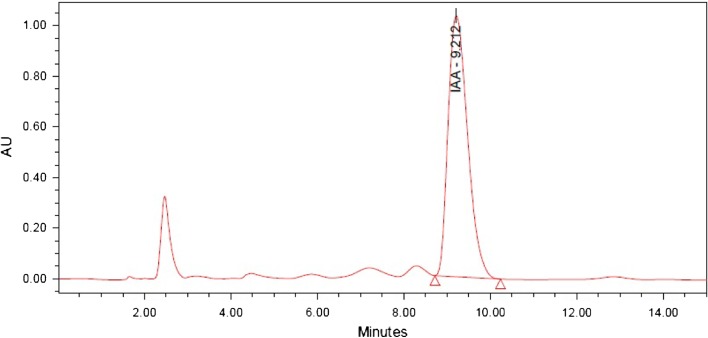
HPLC chromatogram for the extract from isolate ClB2 carried out using water and methanol, both containing 0.5 % acetic acid in the ratio 60:40 on reversed phase C18 column with a flow rate 1 mL min^−1^ and detected under UV at 280 nm

The culture supernatant of CB1 and CB2 were found to have the presence of IAA by conducting the preliminary screening using Salkowski reagent and this was further confirmed by TLC and HPLC. In the TLC analysis, the pure IAA and the extracts were found to have *R*_f_ value of around 0.62. The result was finally confirmed by HPLC where the pure IAA produced a peak at 9.3 min retention time (Fig. 2) and the crude extract had a predominant peak at comparable retention time (Figs. 3, 4). Thus, the result of HPLC confirmed the production of indole 3 acetic acid by the bacterial isolates ClB1 and ClB2 (*Paenibacillus* sp.) from turmeric.

Some endophytic microorganisms have the potential to synthesize IAA. For the microbial synthesis of IAA in tryptophan-dependent route, tryptophan is used as the precursor. There are different pathways which can lead to tryptophan-dependent microbial production of IAA. The various pathways for IAA biosynthesis include tryptophol, tryptamine, indole-3-pyruvic acid and indole-3-acetamide pathways (Gravel et al. 2007). The presence of *Paenibacillus* sp. as endophyte and its ability to produce IAA have already been reported from diverse plants (Lebuhn et al. 1997; Timmusk and Wagner 1999). Acuña et al. (2011) reported the increase in the production of IAA by tenfold in the low-nutrient (diluted) LB medium than in nutrient-rich medium by *Paenibacillus* sp. However, the presence of *Paenibacillus* sp. as endophytic bacteria from turmeric and their ability to synthesize IAA have not yet been reported. This makes the present finding much more interesting. Thus, two strains of *Paenibacillus* sp. identified in the study can have important growth-regulating role in the rhizome of turmeric. The strains may also have unique features to survive in the specific chemical environment of the rhizome.

In conclusion, the results of this study demonstrated that, (1) two different strains of *Paenibacillus* sp. are endophytically associated with the rhizome of *Curcuma longa* and (2) both the endophytic *Paenibacillus* sp. strains (ClB1 and ClB2) have the ability to form IAA as confirmed by HPLC analysis. Hence this isolate can be considered as having possible growth-promoting effect in *Curcuma longa*.
